# Existence of Multiple Solutions for a Quasilinear Biharmonic Equation

**DOI:** 10.1155/2014/370494

**Published:** 2014-10-28

**Authors:** Wen-Wu Pan, Cheng-En Yu

**Affiliations:** Department of Science, Sichuan University of Science and Engineering, Zigong 643000, China

## Abstract

Using three critical points theorems, we prove the existence of at least three solutions for a quasilinear biharmonic equation.

## 1. Introduction

In this paper, we show the existence of at least three weak solutions for the Navier boundary value problem

(1) $$\begin{array}{c} \Delta^{2}u-\mathrm{div}\left(\nabla u\right)=\lambda f\left(x,u\right)+g\left(u\right)\;\text{in}\;\Omega ,\\ u=\Delta u=0\;\mathrm{on}\partial \Omega , \end{array}$$

where *Ω* ⊂ *ℝ* ^*N*^  (4 > *N* ≥ 1) is a nonempty bounded open set with a sufficient smooth boundary ∂*Ω*, *λ* > 0, *f* : *Ω* × *ℝ* → *ℝ* is an *L* ^1^-Carathéodory function, and *g* : *ℝ* → *ℝ* is a Lipschitz continuous function with Lipschitz constant *L* > 0; that is,

(2) $$\begin{array}{c} \left|g\left(t_{1}\right)-g\left(t_{2}\right)\right|\leq L\left|t_{1}-t_{2}\right| \end{array}$$

for every *t*_1_, *t*_2_ ∈ *ℝ* and *g*(0) = 0.

Motivated by the fact that such problems are used to describe a large class of physical phenomena, many authors looked for existence and multiplicity of solutions for forth-order nonlinear equations. For an overview on this subject, we cite the papers [1–23]. For instance, when *N* = 1, in [22], Liu and Li, using Ricceri's three critical points theorem [24], established the existence of at least three weak solutions for the following problem:

(3) $$\begin{array}{c} u^{iv}+\alpha u^{\prime \prime }+\beta u=\lambda f\left(x,u\right)\;x\in \left(a,b\right),\\ u\left(a\right)=u\left(b\right)=u^{\prime \prime }\left(a\right)=u^{\prime \prime }\left(b\right)=0, \end{array}$$

where *α*, *β* are real constants and *λ* is a positive parameter and *f* : [*a*, *b*] × *ℝ* → *ℝ* is a *L* ^2^-Carathéodory function. Later, some authors generalized this type of equation (see [1–5, 10, 12, 15, 19]). When *N* > 1, Liu and Su [21] also used Ricceri's three critical points theorem [24] to established the existence of at least three weak solutions for the following problem:

(4) $$\begin{array}{c} \Delta \left({\left|\Delta u\right|}^{p-2}\Delta u\right)-\mathrm{div}\left({\left|\nabla u\right|}^{p-2}\nabla u\right)=\lambda f\left(x,u\right)\;\text{in}\;\Omega ,\\ u=\Delta u=0\;\mathrm{on}\;\partial \Omega , \end{array}$$

where *Ω* ⊂ *ℝ* ^*N*^  (*N* ≥ 1) is a nonempty bounded open set with a sufficient smooth boundary ∂*Ω*, *p* > max⁡⁡{1, *N*/2}, *λ* > 0, and *f* : *Ω* × *ℝ* → *ℝ* is an *L* ^1^-Carathéodory function. After that some authors used different critical point theorems to get one nontrivial, at least three, and infinitely many solutions (see [6–8, 16, 18]). Elliptic systems were also considered by [9, 11, 13, 14, 17, 20, 23].

The goal of the present paper is to establish some new criteria for (1) to have at least three weak solutions (Theorems 4 and 5). Our analysis is mainly based on recent critical point theorems that are contained in Theorems 2 and 3. In fact, employing rather different three critical points theorems, under different assumptions on the nonlinear term *f*, we obtain the exact collections of *λ* for which (1) admits at least three weak solutions in the space *W* ^2,2^(*Ω*)∩*W*_0_^1,2^(*Ω*).

A special case of our main results is the following theorem.

**Theorem 1 .**

Let *g* : *ℝ* → *ℝ* be a Lipschitz continuous function with the Lipschitz constant *L* > 0 and *g*(0) = 0 such that *L* < 1/*K* ^2^ *m*(*Ω*), where *K* is defined by (17). Let *f* : *ℝ* → *ℝ* be a continuous function and put *F*(*t*) = ∫_0_^*t*^ *f*(*ξ*)*dξ* for each *t* ∈ *ℝ*. Assume that *F*(*d*) > 0 for some *d* > 0 and *F*(*ξ*) ≥ 0 in [0, *d*] and

(5) $$\begin{array}{c} \underset{\xi \to 0}{\lim\inf}\frac{F\left(\xi \right)}{\xi^{2}}=0,\;\;\underset{\left|\xi \right|\to +\infty }{\lim\sup}\frac{F\left(\xi \right)}{\xi^{2}}=0. \end{array}$$

Then, there is *λ* ^*^ > 0 such that for each *λ* > *λ* ^*^ the problem

(6) $$\begin{array}{c} \Delta^{2}u-\mathrm{div}\left(\nabla u\right)=\lambda f\left(u\right)+g\left(u\right)\;in\;\Omega ,\\ u=\Delta u=0\;on\;\partial \Omega \end{array}$$

admits at least three weak solutions.

## 2. Preliminaries

First we here recall for the reader's convenience our main tools to prove the results. The first result has been obtained in [25] and the second one in [26].

**Theorem 2 (see [25, Theorem 3.1]).**

Let *X* be a separable and reflexive real Banach space, Φ : *X* → *ℝ* a nonnegative continuously Gâteaux differentiable and sequentially weakly lower semicontinuous functional whose Gâteaux derivative admits a continuous inverse on *X* ^*^, and Ψ : *X* → *ℝ* a continuously Gâteaux differentiable functional whose Gâteaux derivative is compact. Assume that there exists *x*_0_ ∈ *X* such that Φ(*x*_0_) = Ψ(*x*_0_) = 0 and that

(7) $$\begin{array}{c} \underset{||x||\to +\infty }{\lim}\left(\Phi \left(x\right)-\lambda \Psi \left(x\right)\right)=+\infty \;\forall \lambda \in [0,+\infty [. \end{array}$$

Further, assume that there are *r* > 0, *x*_1_ ∈ *X*, such that *r* < Φ(*x*_1_) and

(8) $$\begin{array}{c} \underset{x\in {\bar{\Phi^{-1}(]-\infty ,r[)}}^{w}}{\sup}\Psi \left(x\right)<\frac{r}{r+\Phi \left(x_{1}\right)}\Psi \left(x_{1}\right); \end{array}$$

here ${\bar{\Phi^{-1}(]-\infty ,r[)}}^{w}$ denotes the closure of Φ^−1^(] − *∞*, *r*[) in the weak topology. Then, for each

(9) λ∈Λ1∶=Φ(x1)sup⁡x∈Φ−1(]−∞,r[)¯w]Φ(x1)Ψ(x1)−sup⁡x∈Φ−1(]−∞,r[)¯wΨ(x),    rsup⁡x∈Φ−1(]−∞,r[)¯wΨ(x)[rsup⁡x∈Φ−1(]−∞,r[)¯w,

the equation

(10) $$\begin{array}{c} \Phi^{\prime }\left(u\right)-\lambda \Psi^{\prime }\left(u\right)=0 \end{array}$$

has at least three solutions in *X* and, moreover, for each *h* > 1, there exists an open interval

(11) $$\begin{array}{c} \Lambda_{2}\subseteq \left[0,\frac{hr}{r\left(\Psi \left(x_{1}\right)/\Phi \left(x_{1}\right)\right)-\sup_{x\in {\bar{\Phi^{-1}(-\infty ,r)}}^{w}}^{}\Psi \left(x\right)}\right] \end{array}$$

and a positive real number *σ* such that, for each *λ* ∈ Λ_2_, (10) has at least three solutions in *X* whose norms are less than *σ*.

**Theorem 3 (see [26, Theorem 3.6]).**

Let *X* be a reflexive real Banach space; let Φ : *X* → *ℝ* be a sequentially weakly lower semicontinuous, coercive, and continuously Gâteaux differentiable whose Gâteaux derivative admits a continuous inverse on *X* ^*^, and let Ψ : *X* → *ℝ* be a sequentially weakly upper semicontinuous and continuously Gâteaux differentiable functional whose Gâteaux derivative is compact. Assume that there exist *r* ∈ *ℝ* and *u*_1_ ∈ *X* with 0 < *r* < Φ(*u*_1_), such that

(A1) sup⁡_*u*∈Φ^−1^(]−*∞*,*r*])_Ψ(*u*) < *r*(Ψ(*u*_1_)/Φ(*u*_1_));
(A2) for each *λ* ∈ Λ_*r*_∶ = ]Φ(*u*_1_)/Ψ(*u*_1_), *r*/sup⁡_*u*∈Φ^−1^(]−*∞*,*r*])_Ψ(*u*)[ the functional Φ − *λ*Ψ is coercive.

Then, for each *λ* ∈ Λ_*r*_, the functional Φ − *λ*Ψ has at least three distinct critical points in *X*.

Let *f* : *Ω* × *ℝ* → *ℝ* be an *L* ^1^-Carathéodory function and let *g* : *ℝ* → *ℝ* be a Lipschitz continuous function with the Lipschitz constant *L* > 0; that is,

(12) $$\begin{array}{c} \left|g\left(t_{1}\right)-g\left(t_{2}\right)\right|\leq L\left|t_{1}-t_{2}\right| \end{array}$$

for every *t*_1_, *t*_2_ ∈ *ℝ*, and *g*(0) = 0.

Put

(13) $$\begin{array}{c} F\left(x,t\right)∶=\overset{t}{\underset{0}{\int }}f\left(x,\xi \right)d\xi ,\;\;G\left(t\right)∶=-\overset{t}{\underset{0}{\int }}g\left(\xi \right)d\xi \end{array}$$

for all *x* ∈ *Ω* and *t* ∈ *ℝ*. Denote

(14) $$\begin{array}{c} X∶=W^{2,2}\left(\Omega \right)\cap W_{0}^{1,2}\left(\Omega \right); \end{array}$$

the usual norm in *X* is defined by

(15) $$\begin{array}{c} ||u||∶={\left(\int_{\Omega }{\left|\Delta u(x)\right|}^{2}+{\left|\nabla u\left(x\right)\right|}^{2}dx\right)}^{1/2}. \end{array}$$

Note that *X* is a separable and reflexive real Banach space.

We say that a function *u* ∈ *X* is a* weak solution* of problem (1) if

(16) ∫ΩΔu(x)Δv(x)dx+∫Ω∇u(x)·∇v(x)dx  =λ∫Ωf(x,u(x))v(x)dx−∫Ωg(u(x))v(x)dx=0,

for all *v* ∈ *X*.

It is well known that (*X*, ||·||) is embedded in (*C* ^0^(*Ω*), ||·||_*∞*_) and

(17) $$\begin{array}{c} {||u||}_{\infty }\leq K||u|| \end{array}$$

for all *u* ∈ *X*. Since *N* < 4, one has *K* < +*∞*.

Suppose that the Lipschitz constant *L* > 0 of the function *g* satisfies *L* < 1/*K* ^2^ *m*(*Ω*). For other basic notations and definitions, we refer the reader to [27–29].

## 3. Main Results

Our main results are the following theorems.

**Theorem 4 .**

Assume that there exist a function *w* ∈ *X*, a positive function *a* ∈ *L* ^1^, and two positive constants *r* and *γ* with *γ* < *p* such that

(A1) ||*w*||^2^ > 2*r*/(1 − *K* ^2^ *Lm*(*Ω*));
(A2) $\int_{\Omega }\sup_{|t|\leq K\sqrt{2r/\left(1-LK^{2}m(\Omega )\right)}}F(x,t)dx<r\left(\int_{\Omega }F(x,w(x))dx/\left(r+\left(\left(1+K^{2}Lm(\Omega )\right)/2\right){||w||}^{2}\right)\right)$;
(A3) *F*(*x*, *t*) ≤ *a*(*x*)(1 + |*t*|^*γ*^) for a.e. *x* ∈ *Ω* and for all *t* ∈ *ℝ*.

Then, for each *λ* in

(18) Λ1:=(1)sup⁡|t|≤K2r/(1−LK2m(Ω))](((1+K2Lm(Ω))/2)||w||2)    ×(sup⁡|t|≤K2r/(1−LK2m(Ω))∫ΩF(x,w(x))dx      −∫Ωsup⁡|t|≤K2r/(1−LK2m(Ω))F(x,t)dx)−1,      r∫Ωsup⁡|t|≤K2r/(1−LK2m(Ω))F(x,t)dx[r∫Ωsup⁡|t|≤K2r/(1−LK2m(Ω))F(x,t)dx,

problem (1) admits at least three weak solutions in *X* and, moreover, for each *h* > 1, there exists an open interval

(19) Λ2⊆[−∫Ωsup⁡|t|≤K2r/(1−LK2m(Ω))F(x,t)dx)−10,(hr)(sup⁡|t|≤K2r/(1−LK2m(Ω))2r(1+K2Lm(Ω))||w||2∫ΩF(x,w(x))dx        −∫Ωsup⁡|t|≤K2r/(1−LK2m(Ω))F(x,t)dx)−1]

and a positive real number *σ* such that, for each *λ* ∈ Λ_2_, the problem (1) admits at least three weak solutions in *X* whose norms are less than *σ*.

**Theorem 5 .**

Assume that there exists a function *w* ∈ *X* and a positive constant *r* such that

(B1) ||*w*||^2^ > 2*r*/(1 − *K* ^2^ *Lm*(*Ω*));
(B2) $\int_{\Omega }\sup_{|t|\leq K\sqrt{2r/\left(1-LK^{2}m(\Omega )\right)}}F(x,t)dx/r<\left(2/\left(1+K^{2}Lm(\Omega )\right)\right)\left(\int_{\Omega }F(x,w(x))dx/{||w||}^{2}\right)$;
(B3) $2K^{2}/\left(1-K^{2}Lm(\Omega )\right){\lim\sup}_{|t|\to +\infty }F(x,t)/t^{2}<\left(\int_{\Omega }\sup_{|t|\leq K\sqrt{2r/\left(1-LK^{2}m(\Omega )\right)}}F(x,t)dx\right)/r$.

Then, for each

(20) λ∈r∫Ωsup⁡|t|≤K2r/(1−LK2m(Ω))F(x,t)dx]1+K2Lm(Ω)2||w||2∫ΩF(x,w(x))dx, r∫Ωsup⁡|t|≤K2r/(1−LK2m(Ω))F(x,t)dx[r∫Ωsup⁡|t|≤K2r/(1−LK2m(Ω))F(x,t)dx,

problem (1) admits at least three weak solutions.

Let us give particular consequences of Theorems 4 and 5 for a fixed test function *w*. Now, fix *x* ^0^ ∈ *Ω* and pick *γ* with *γ* > 0 such that *B*(*x* ^0^, *γ*) ⊂ *Ω* where *B*(*x* ^0^, *γ*) denotes the ball with center at *x* ^0^ and radius of *γ*. Put

(21) Q=∫B(x0,γ)∖B(x0,γ/2)|12γ3|x−x0|l−24γ2l+9γl|x−x0||2dx,R=πN/2Γ(N/2)∫(γ/2)2γ2|12(N+1)γ3t+9(N−1)γ1t−24Nγ2|2×tN/2−1dt,

(22) $$\begin{array}{c} \theta =K{\left(R+Q\right)}^{1/2}, \end{array}$$

where *l* = (∑_*i*=1_^*N*^ *x*_*i*_^2^)^1/2^, $\left|x-x^{0}\right|=\sqrt{\sum_{i=1}^{N}{\left(x_{i}-x_{i}^{0}\right)}^{2}}$, and *m*(*Ω*) denotes the volume of *Ω*.

**Corollary 6 .**

Assume that there exist a positive function *a* ∈ *L* ^1^ and three positive constants *c*, *d*, and *γ* with *c* < *θd* and *γ* < 2 such that Assumption (A3) in Theorem 4 holds. Furthermore, suppose that

(A4) *F*(*x*, *t*) ≥ 0 for a.e. *x* ∈ *Ω*∖*B*(*x* ^0^, *γ*/2) and all *t* ∈ [0, *d*];
(A5) ∫_*Ω*_sup⁡_*t*∈[−*c*,*c*]_ *F*(*x*, *t*)*dx* < (1 − *K* ^2^ *Lm*(*Ω*))*c* ^2^(∫_*B*(*x*^0^,*γ*/2)_ *F*(*x*, *d*)*dx*/((1 − *K* ^2^ *Lm*(*Ω*))*c* ^2^+(1 + *K* ^2^ *Lm*(*Ω*))(*θd*)^2^)).

Then, for each *λ* in

(23) Λ1′∶=1−K2Lm(Ω)2c22K2∫Ωsup⁡t∈[−c,c]F(x,t)dx]1+K2Lm(Ω)2      ×(θd)2(−∫Ωsup⁡t∈[−c,c]F(x,t)dx)2K2(∫B(x0,γ/2)F(x,d)dx           −∫Ωsup⁡t∈[−c,c]F(x,t)dx))−1,     1−K2Lm(Ω)2c22K2∫Ωsup⁡t∈[−c,c]F(x,t)dx[1−K2Lm(Ω)2c22K2∫Ωsup⁡t∈[−c,c]F(x,t)dx,

problem (1) admits at least three weak solutions in *X* and, moreover, for each *h* > 1, there exist an open interval

(24) Λ2′⊆[0,1−K2Lm(Ω)∫Ωsup⁡t∈[−c,c]2  ×(h/2)(c/K)2(c/θd)2∫B(x0,γ/2)F(x,d)dx−∫Ωsup⁡t∈[−c,c]F(x,t)dx]

and a positive real number *σ* such that, for each *λ* ∈ Λ_2_′, problem (1) admits at least three weak solutions in *X* whose norms are less than *σ*.

**Proof**

We claim that all the assumptions of Theorem 4 are fulfilled with *w* given by

(25) w(x)={0 for  x∈Ω∖B(x0,γ),d(4γ3|x−x0|3−12γ2|x−x0|2aaa+9γ|x−x0|−1)  for  x∈B(x0,γ)∖B(x0,γ/2),d for  x∈B(x0,γ/2),

and *r*∶ = ((1 − *K* ^2^ *Lm*(*Ω*))/2)(*c*/*K*)^2^. It is easy to verify that *w* ∈ *W* ^2,2^(*Ω*)∩*W*_0_^1,2^(*Ω*), and, in particular, one has

(26) $$\begin{array}{c} {||w||}^{2}=\left(R+Q\right)d^{2}, \end{array}$$

and consequently from (22) we see that

(27) $$\begin{array}{c} ||w||=\frac{\theta d}{K}. \end{array}$$

Thus, (A1) holds. Since 0 ≤ *w*(*x*) ≤ *d* for each *x* ∈ *Ω*, the condition (A4) ensures that

(28) ∫Ω∖B(x0,γ)F(x,w(x))dx  +∫B(x0,γ)∖B(x0,γ/2)F(x,w(x))dx≥0,

so, from (A5),

(29) ∫Ωsup⁡t∈[−c,c]F(x,t)dx <(1−K2Lm(Ω))c2  ×∫B(x0,γ/2)F(x,d)dx(1−K2Lm(Ω))c2+(1+K2Lm(Ω))(θd)2 =(1−K2Lm(Ω))c212K2  ×((∫B(x0,γ/2)F(x,d)dx)    ×(12K2(1−K2Lm(Ω))c2      +12K2(1+K2Lm(Ω))(θd)2)−1) =r∫ΩF(x,w(x))dxr+((1+K2Lm(Ω))/2)||w||2,

and thus (A2) holds. Next notice that

(30) ((1+K2Lm(Ω))/2)||w||2∫ΩF(x,w(x))dx−∫Ωsup⁡|t|≤K2r/(1−LK2m(Ω))F(x,t)dx  ≤(1+K2Lm(Ω)(θd)2)/2K2∫B(x0,γ/2)F(x,d)dx−∫Ωsup⁡t∈[−c,c]F(x,t)dx,r∫Ωsup⁡|t|≤K2r/(1−LK2m(Ω))F(x,t)dx  =(1−K2Lm(Ω))c22K2∫Ωsup⁡t∈[−c,c]F(x,t)dx.

In addition note that

(31) (1+K2Lm(Ω)(θd)2)/2K2∫B(x0,γ/2)F(x,d)dx−∫Ωsup⁡t∈[−c,c]F(x,t)dx<(1+K2Lm(Ω)d(θ)22K2) ×(((1−K2Lm(Ω))c2(1−K2Lm(Ω))c2+(1+K2Lm(Ω))(θd)2−1)   ×∫Ωsup⁡t∈[−c,c]F(x,t)dx((1−K2Lm(Ω))c2(1−K2Lm(Ω))c2+(1+K2Lm(Ω))(θd)2−1))−1=(1−K2Lm(Ω))c22K2∫Ωsup⁡t∈[−c,c]F(x,t)dx.

Finally note that

(32) (hr)(sup⁡|t|≤K2r/(1−LK2m(Ω))2r(1+K2Lm(Ω))||w||2∫ΩF(x,w(x))dx   −∫Ωsup⁡|t|≤K2r/(1−LK2m(Ω))F(x,t)dx)−1  ≤1−K2Lm(Ω)2   ×(h2(cK)2)(∫Ωsup⁡t∈[−c,c](cθd)2∫B(x0,γ/2)F(x,d)dx           −∫Ωsup⁡t∈[−c,c]F(x,t)dx)−1,

and, taking into account that Λ_1_′⊆Λ_1_ and Λ_2_⊆Λ_2_′, we have the desired conclusion directly from Theorem 4.

**Corollary 7 .**

Assume that there exist two positive constants *c* and *d* with *c* < *θd* such that the assumption (A4) in Corollary 6 holds. Furthermore, suppose that

(B4) ∫_*Ω*_sup⁡_*t*∈[−*c*,*c*]_ *F*(*x*, *t*)*dx*/(1 − *K* ^2^ *Lm*(*Ω*))*c* ^2^ < ∫_*B*(*x*^0^,*γ*/2)_ *F*(*x*, *d*)*dx*/(1 + *K* ^2^ *Lm*(*Ω*))(*θd*)^2^;
(B5) limsup⁡_|*t*|→+*∞*_ *F*(*x*, *t*)/*t* ^2^ < ∫_*Ω*_sup⁡_*t*∈[−*c*,*c*]_ *F*(*x*, *t*)*dx*/*c* ^2^.

Then, for each

(33) λ∈(θd)2∫B(x0,γ/2)F(x,d)dx]1+K2Lm(Ω)212K2(θd)2∫B(x0,γ/2)F(x,d)dx, 1−K2Lm(Ω)2c22K2∫Ωsup⁡t∈[−c,c]F(x,t)dx[(θd)2∫B(x0,γ/2)F(x,d)dx,

problem (1) admits at least three weak solutions.

**Proof**

All the assumptions of Theorem 5 are fulfilled by choosing *w* as given in (25) and *r*∶ = ((1 − *K* ^2^ *Lm*(*Ω*))/2)(*c*/*K*)^2^ and bearing in mind that

(34) $$\begin{array}{c} ||w||=\frac{\theta d}{K} \end{array}$$

and recalling

(35) ∫Ω∖B(x0,γ)F(x,w(x))dx +∫B(x0,γ)∖B(x0,γ/2)F(x,w(x))dx≥0.

Hence, by applying Theorem 5, we have the conclusion.

**Proof of Theorem 1.**

Fix *λ* > *λ* ^*^∶ = *m*(*Ω*)(1 + *K* ^2^ *Lm*(*Ω*))(*θd*)^2^/*m*(*B*(*x* ^0^, *γ*))*F*(*d*) for some *d* > 0. Since

(36) $$\begin{array}{c} \underset{\xi \to 0}{\lim\inf}\frac{F\left(\xi \right)}{\xi^{2}}=0, \end{array}$$

there is {*c*_*m*_}_*m*∈*ℕ*_⊆  ]0, +*∞*[ such that lim⁡_*m*→+*∞*_ *c*_*m*_ = 0 and

(37) $$\begin{array}{c} \underset{m\to +\infty }{\lim}\frac{\sup_{|\xi |\leq c_{m}}F\left(\xi \right)}{c_{m}}=0. \end{array}$$

In fact, one has

(38) $$\begin{array}{c} \underset{m\to +\infty }{\lim}\frac{\sup_{|\xi |\leq c_{m}}F\left(\xi \right)}{c_{m}}=\underset{m\to +\infty }{\lim}\frac{F\left(\xi_{c_{m}}\right)}{\xi_{c_{m}}^{2}}\cdot \frac{\xi_{c_{m}}^{2}}{c_{m}}=0, \end{array}$$

where *F*(*ξ*_*c*_*m*__) = sup⁡_|*ξ*|≤*c*_*m*__ *F*(*ξ*). Hence, there is $\bar{c}>0$ such that

(39) sup⁡|ξ|≤c¯F(ξ)c¯2<min⁡{m(B(x0,γ))(1−KpLm(Ω))F(d)m(Ω)(1+K2Lm(Ω))(θd)2, (1−K2Lm(Ω))λ}

and $\bar{c}<\theta d$. From Corollary 7 we have the desired conclusion.

## 4. Proofs

**Proof of Theorem 4.**

Our aim is to apply Theorem 2 to our problem. To this end, for each *u* ∈ *X*, we let the functionals Φ, Ψ : *X* → *ℝ* be defined by

(40) $$\begin{array}{c} \Phi \left(u\right)∶=\frac{1}{2}{||u||}^{2}+\int_{\Omega }G\left(u\left(x\right)\right)dx,\\ \Psi \left(u\right)∶=\int_{\Omega }F\left(x,u\left(x\right)\right)dx, \end{array}$$

and put

(41) $$\begin{array}{c} I_{\lambda }\left(u\right)∶=\Phi \left(u\right)-\lambda \Psi \left(u\right)\;\forall u\in X. \end{array}$$

The functionals Φ and Ψ satisfy the regularity assumptions of Theorem 2. Indeed, by standard arguments, we have that Φ is Gâteaux differentiable and sequentially weakly lower semicontinuous and its Gâteaux derivative at the point *u* ∈ *X* is the functional Φ′(*u*) ∈ *X* ^*^, given by

(42) Φ′(u)(v)=∫ΩΔu(x)Δv(x)dx+∫Ω∇u(x)·∇v(x)dx−∫Ωg(u(x))v(x)dx,

for every *v* ∈ *X*. Furthermore, the differential Φ′ : *X* → *X* ^*^ is a Lipschitzian operator. Indeed, for any *u*, *v* ∈ *X*, there holds

(43) ||Φ′(u)−Φ′(v)||X∗  =sup⁡||w||≤1|(Φ′(u)−Φ′(v),w)|  ≤sup⁡||w||≤1|(u−v,w)|    +sup⁡||w||≤1∫Ω|g(u(x))−g(v(x))||w(x)|dx  ≤sup⁡||w||≤1||u−v||||w||    +sup⁡||w||≤1(∫Ω|g(u(x))−g(v(x))|2)1/2    ×(∫Ω|w(x)|2)1/2.

Recalling that *g* is Lipschitz continuous and the embedding *X*↪*L* ^2^(*Ω*) is compact, the claim is true. In particular, we derive that Φ is continuously differentiable. The inequality (17) yields for any *u*, *v* ∈ *X* the estimate

(44) (Φ′(u)−Φ′(v),u−v)  =(u−v,u−v)−∫Ω(g(u(x))−g(v(x)))           ×(u(x)−v(x))dx  ≥(1−K2Lm(Ω))||u−v||2.

By the assumption *L* < 1/*K* ^2^ *m*(*Ω*), it turns out that Φ′ is a strongly monotone operator. So, by applying Minty-Browder theorem [29, Theorem 26.A],  Φ′ : *X* → *X* ^*^ admits a Lipschitz continuous inverse. On the other hand, the fact that *X* is compactly embedded into *C* ^0^(*Ω*) implies that the functional Ψ is well defined, continuously Gâteaux differentiable, and with compact derivative, whose Gateaux derivative at the point *u* ∈ *X* is given by

(45) $$\begin{array}{c} \Psi^{\prime }\left(u\right)\left(v\right)=\int_{\Omega }f\left(x,u\left(x\right)\right)v\left(x\right)dx, \end{array}$$

for every *v* ∈ *X*. Note that the weak solutions of (1) are exactly the critical points of *I*_*λ*_. Also, since *g* is Lipschitz continuous and satisfies *g*(0) = 0, we have from (17) that

(46) $$\begin{array}{c} \frac{1-K^{2}Lm\left(\Omega \right)}{2}{||u||}^{2}\leq \Phi \left(u\right)\leq \frac{1+K^{2}Lm\left(\Omega \right)}{2}{||u||}^{2}, \end{array}$$

for all *u* ∈ *X*, and so Φ is coercive.

Furthermore from (A3) for any fixed *λ* ∈ [0, +*∞*[, using (46), taking (17) into account, we have

(47) Φ(u)−λΨ(u)  =12||u||2+∫ΩG(u(x))dx−λ∫ΩF(x,u(x))dx  ≥1−K2Lm(Ω)2||u||2−λ∫Ωa(x)(1+|u(x)|γ)dx  ≥1−K2Lm(Ω)2||u||2−λ||a||L1(Ω)(m(Ω)+Kγ||u||γ),

and so

(48) $$\begin{array}{c} \underset{||u||\to +\infty }{\lim}\left(\Phi \left(u\right)-\lambda \Psi \left(u\right)\right)=+\infty . \end{array}$$

Also according to (A1) we achieve Φ(*w*) > *r*. From the definition of Φ and by using (46) we have

(49) Φ−1(]−∞,r[)  ={u∈X:Φ(u)<r}  ⊆{u∈X:||u||<2r1−LK2m(Ω)}  ⊆{u∈X:|u(x)|<K2r1−LK2m(Ω)  ∀x∈Ω}.

So, we obtain

(50) $$\begin{array}{c} \underset{u\in {\bar{\Phi^{-1}(]-\infty ,r[)}}^{w}}{\sup}\Psi \left(u\right)\leq \int_{\Omega }\underset{\left|t\right|\leq K\sqrt{2r/\left(1-LK^{2}m(\Omega )\right)}}{\sup}F\left(x,t\right)dx. \end{array}$$

Therefore, from (A2) and (46), we have

(51) sup⁡u∈Φ−1(]−∞,r[)¯wΨ(u)  ≤∫Ωsup⁡|t|≤K2r/(1−LK2m(Ω))F(x,t)dx  <rr+((1+K2Lm(Ω))/2)||w||2∫ΩF(x,w(x))dx  <rr+Φ(w)Ψ(w).

Now, we can apply Theorem 2. Note that, for each *x* ∈ *Ω*,

(52) Φ(w)Ψ(w)−sup⁡u∈Φ−1(]−∞,r[)¯wΨ(u)  ≤(1+K2Lm(Ω)2||w||2)   ×(sup⁡|t|≤K2r/(1−LK2m(Ω))∫ΩF(x,w(x))dx     −∫Ωsup⁡|t|≤K2r/(1−LK2m(Ω))F(x,t)dx)−1,rsup⁡u∈Φ−1(]−∞,r[)¯wΨ(u)  ≥r∫Ωsup⁡|t|≤K2r/(1−LK2m(Ω))F(x,t)dx.

Note also that (A2) implies

(53) ((1+K2Lm(Ω))/2)||w||2∫ΩF(x,w(x))dx−∫Ωsup⁡|t|≤K2r/(1−LK2m(Ω))F(x,t)dx  <(1+K2Lm(Ω)2||w||2)   ×(sup⁡2r/(1−LK2m(Ω))(r+((1+K2Lm(Ω))/2)||w||2r−1)     ×∫Ωsup⁡|t|≤K2r/(1−LK2m(Ω))F(x,t)dx)−1  =r∫Ωsup⁡|t|≤K2r/(1−LK2m(Ω))F(x,t)dx.

Also,

(54) hrr(Ψ(w)/Φ(w))−sup⁡u∈Φ−1(−∞,r)¯wΨ(u)  ≤(hr)(∫Ωsup⁡2r/1−LK2m(Ω)2r(1+K2Lm(Ω))||w||2∫ΩF(x,w(x))dx      −∫Ωsup⁡|t|≤K2r/(1−LK2m(Ω))F(x,t)dx)−1=ρ.

From (A2) it follows that

(55) 2r(1+K2Lm(Ω))||w||2∫ΩF(x,w(x))dx   −∫Ωsup⁡|t|≤K2r/(1−LK2m(Ω))F(x,t)dx     >(2r(1+K2Lm(Ω))||w||2       −rr+((1+K2Lm(Ω))/2)||w||2)   ×∫ΩF(x,w(x))dx  ≥(2r(1+K2Lm(Ω))||w||2−2r(1+K2Lm(Ω))||w||2)   ×∫ΩF(x,w(x))dx=0,

since ∫_*Ω*_ *F*(*x*, *w*(*x*))*dx* ≥ 0 (note *F*(*x*, 0) = 0 so $\int_{\Omega }\sup_{|t|\leq K\sqrt{2r/\left(1-LK^{2}m(\Omega )\right)}}F(x,t)dx\geq 0$ and now apply (A2)). Now with *x*_0_ = 0 and *x*_1_ = *w* from Theorem 2 (note Ψ(0) = 0) it follows that, for each *λ* ∈ Λ_1_, the problem (1) admits at least three weak solutions and there exist an open interval Λ_2_⊆[0, *ρ*] and a real positive number *σ* such that, for each *λ* ∈ Λ_2_, the problem (1) admits at least three weak solutions whose norms in *X* are less than *σ*. Thus, the conclusion is achieved.

**Proof of Theorem 5.**

To apply Theorem 3 to our problem, we take the functionals Φ, Ψ : *X* → *ℝ* as given in the proof of Theorem 4. Let us prove that the functionals Φ and Ψ satisfy the conditions required in Theorem 3. The regularity assumptions on Φ and Ψ, as requested in Theorem 3, hold. According to (B1) we deduce Φ(*w*) > *r*. From the definition of Φ we have

(56) Φ−1(]−∞,r[)  ⊆{u∈X:|u(x)|<K2r1−LK2m(Ω)  ∀x∈Ω},

and it follows that

(57) $$\begin{array}{c} \underset{u\in \Phi^{-1}(]-\infty ,r[)}{\sup}\Psi \left(u\right)\leq \int_{\Omega }\underset{\left|t\right|\leq K\sqrt{2r/\left(1-LK^{2}m(\Omega )\right)}}{\sup}F\left(x,t\right)dx. \end{array}$$

Therefore, due to assumption (B2), we have

(58) sup⁡u∈Φ−1(]−∞,r[)Ψ(u)r  ≤∫Ωsup⁡|t|≤K2r/(1−LK2m(Ω))F(x,t)dxr  <21+K2Lm(Ω)∫ΩF(x,w(x))dx||w||p  ≤Ψ(w)Φ(w).

Furthermore, from (B3) there exist two constants *η*, *ϑ* ∈ *ℝ* with

(59) $$\begin{array}{c} \eta <\frac{\int_{\Omega }\sup_{|t|\leq K\sqrt{2r/\left(1-LK^{2}m(\Omega )\right)}}F\left(x,t\right)dx}{r} \end{array}$$

such that

(60) $$\begin{array}{c} \frac{2K^{2}}{1-K^{2}Lm\left(\Omega \right)}F\left(x,t\right)\leq \eta t^{2}+\vartheta \end{array}$$

for all *x* ∈ *Ω* and all *t* ∈ *ℝ*. Fix *u* ∈ *X*. Then

(61) $$\begin{array}{c} F\left(x,u\left(x\right)\right)\leq \frac{1-K^{2}Lm\left(\Omega \right)}{2K^{2}}\left(\eta {\left|u\left(x\right)\right|}^{2}+\vartheta \right) \end{array}$$

for all *x* ∈ *Ω*. Now, to prove the coercivity of the functional Φ − *λ*Ψ, first we assume that *η* > 0. So, for any fixed

(62) λ∈r∫Ωsup⁡|t|≤K2r/(1−LK2m(Ω))F(x,t)dx]1+K2Lm(Ω)2||w||2∫ΩF(x,w(x))dx, r∫Ωsup⁡|t|≤K2r/(1−LK2m(Ω))F(x,t)dx[r∫Ωsup⁡|t|≤K2r/(1−LK2m(Ω))F(x,t)dx,

using (61), we have

(63) Φ(u)−λΨ(u)  =12||u||2+∫ΩG(u(x))dx−λ∫ΩF(x,u(x))dx  ≥1−K2Lm(Ω)2||u||2−λ1−K2Lm(Ω)2K2   ×(η∫Ω|u(x)|2dx+ϑ)  ≥1−K2Lm(Ω)2   ×(1−ηr∫Ωsup⁡|t|≤K2r/(1−LK2m(Ω))F(x,t)dx)   ×||u||2−1−K2Lm(Ω)2ϑ,

and thus

(64) $$\begin{array}{c} \underset{||u||\to +\infty }{\lim}\left(\Phi \left(u\right)-\lambda \Psi \left(u\right)\right)=+\infty . \end{array}$$

On the other hand, if *η* ≤ 0, clearly we obtain lim⁡_||*u*||→+*∞*_(Φ(*u*) − *λ*Ψ(*u*)) = +*∞*. Both cases lead to the coercivity of functional Φ − *λ*Ψ.

So, assumptions (A1) and (A2) in Theorem 3 are satisfied. Hence, by using Theorem 3, the problem (1) admits at least three distinct weak solutions in *X*.

## References

[1] Afrouzi G. A., Heidarkhani S., O’Regan D. (2011). Existence of three solutions for a doubly eigenvalue fourth-order boundary value problem. *Taiwanese Journal of Mathematics*.

[2] Bonanno G., Di Bella B. (2008). A boundary value problem for fourth-order elastic beam equations. *Journal of Mathematical Analysis and Applications*.

[3] Bonanno G., Di Bella B. (2010). A fourth-order boundary value problem for a Sturm-Liouville type equation. *Applied Mathematics and Computation*.

[4] Bonanno G., Di Bella B. (2011). Infinitely many solutions for a fourth-order elastic beam equation. *Nonlinear Differential Equations and Applications*.

[5] Bonanno G., Di Bella B., O'Regan D. (2011). Non-trivial solutions for nonlinear fourth-order elastic beam equations. *Computers & Mathematics with Applications*.

[6] Candito P., Li L., Livrea R. (2012). Infinitely many solutions for a perturbed nonlinear Navier boundary value problem involving the *p*-biharmonic. *Nonlinear Analysis: Theory, Methods & Applications*.

[7] Candito P., Livrea R. (2010). Infinitely many solution for a nonlinear Navier boundary value problem involving the *p*-biharmonic. *Studia Universitatis Babes-Bolyai Mathematica*.

[8] Candito P., Bisci G. M. (2012). Multiple solutions for a Navier boundary value problem involving the *p*-biharmonic operator. *Discrete and Continuous Dynamical Systems S*.

[9] Graef J. R., Heidarkhani S., Kong L. (2013). Multiple solutions for a class of (*p*
_1_ ⋯ *p*
_*n*_)- biharmonic systems. *Communications on Pure and Applied Analysis*.

[10] Heidarkhani S. (2012). Existence of solutions for a two-point boundary-value problem of a fourth-order Sturm-Liouville type. *Electronic Journal of Differential Equations*.

[11] Heidarkhani S. (2012). Non-trivial solutions for a class of (*p*
_1_,…,*p*
_*n*_)-biharmonic systems with Navier boundary conditions. *Annales Polonici Mathematici*.

[12] Heidarkhani S. (2012). Non-trivial solutions for two-point boundary-value problems of fourth-order Sturm-Liouville type equations. *Electronic Journal of Differential Equations*.

[13] Heidarkhani S. (2012). Three solutions for a class of (*p*
_1_,…,*p*
_*n*_)-biharmonic systems via variational methods. *Thai Journal of Mathematics*.

[14] Heidarkhani S., Tian Y., Tang C. (2012). Existence of three solutions for a class of (*p*
_1_,…,*p*
_*n*_)-biharmonic systems with Navier boundary conditions. *Annales Polonici Mathematici*.

[15] Khalkhali S. M., Heidarkhani S., Razani A. (2012). Infinitely many solutions for a fourth-order boundary-value problem. *Electronic Journal of Differential Equations*.

[16] Li C., Tang C.-L. (2010). Three solutions for a Navier boundary value problem involving the *p*-biharmonic. *Nonlinear Analysis. Theory, Methods & Applications*.

[17] Li L. (2013). Existence of three solutions for a class of Navier quasili near elliptic systems involving the (*p*
_1_,…,*p*
_n_)-biharmonic. *Bulletin of the Korean Mathematical Society*.

[18] Li L., Heidarkhani S. (2012). Existence of three solutions to a double eigenvalue problem for the *p*-biharmonic equation. *Annales Polonici Mathematici*.

[19] Li L., Heidarkhani S. (2013). Multiplicity solutions to a doubly eigenvalue fourth-order equation. *The Journal of the Indian Mathematical Society*.

[20] Li L., Tang C.-L. (2010). Existence of three solutions for (*p*-*q*)-biharmonic systems. *Nonlinear Analysis: Theory, Methods & Applications*.

[21] Liu H., Su N. (2008). Existence of three solutions for a *p*-biharmonic problem. *Dynamics of Continuous, Discrete & Impulsive Systems A: Mathematical Analysis*.

[22] Liu X.-L., Li W.-T. (2007). Existence and multiplicity of solutions for fourth-order boundary value problems with parameters. *Journal of Mathematical Analysis and Applications*.

[23] Massar M., Hssini E. M., Tsouli N. (2012). Infinitely many solutions for class of Navier boundary (*p; q*)-biharmonic systems. *Electronic Journal of Differential Equations*.

[24] Ricceri B. (2000). On a three critical points theorem. *Archiv der Mathematik*.

[25] Bonanno G. (2004). A critical points theorem and nonlinear differential problems. *Journal of Global Optimization*.

[26] Bonanno G., Marano S. A. (2010). On the structure of the critical set of non-differentiable functions with a weak compactness condition. *Applicable Analysis*.

[27] Zeidler E. (1985). *Nonlinear Functional Analysis and Its Applications. III: Variational Methods and Optimization*.

[28] Zeidler E. (1990). *Nonlinear Functional Analysis and Its Applications: II/A: Linear Monotone Operators*.

[29] Zeidler E. (1990). *Nonlinear Functional Analysis and Its Applications II/B*.

